# Modeling behavior in different delay match to sample tasks in one simple network

**DOI:** 10.3389/fnhum.2013.00408

**Published:** 2013-07-30

**Authors:** Yali Amit, Volodya Yakovlev, Shaul Hochstein

**Affiliations:** ^1^Department of Statistics, Chicago UniversityChicago, IL, USA; ^2^Department of Computer Science, Chicago UniversityChicago, IL, USA; ^3^Neurobiology Department, Life Sciences Institute and Safra Center for Brain Research, Hebrew UniversityJerusalem, Israel

**Keywords:** recurrent networks, Hebbian learning, readout mechanism, reset mechanism, memory, forgetting, familiarity, working memory

## Abstract

Delay match to sample (DMS) experiments provide an important link between the theory of recurrent network models and behavior and neural recordings. We define a simple recurrent network of binary neurons with stochastic neural dynamics and Hebbian synaptic learning. Most DMS experiments involve heavily learned images, and in this setting we propose a readout mechanism for match occurrence based on a smaller increment in overall network activity when the matched pattern is already in working memory, and a reset mechanism to clear memory from stimuli of previous trials using random network activity. Simulations show that this model accounts for a wide range of variations on the original DMS tasks, including ABBA tasks with distractors, and more general repetition detection tasks with both learned and novel images. The differences in network settings required for different tasks derive from easily defined changes in the levels of noise and inhibition. The same models can also explain experiments involving repetition detection with novel images, although in this case the readout mechanism for match is based on higher overall network activity. The models give rise to interesting predictions that may be tested in neural recordings.

## Introduction

**Figure 1:**
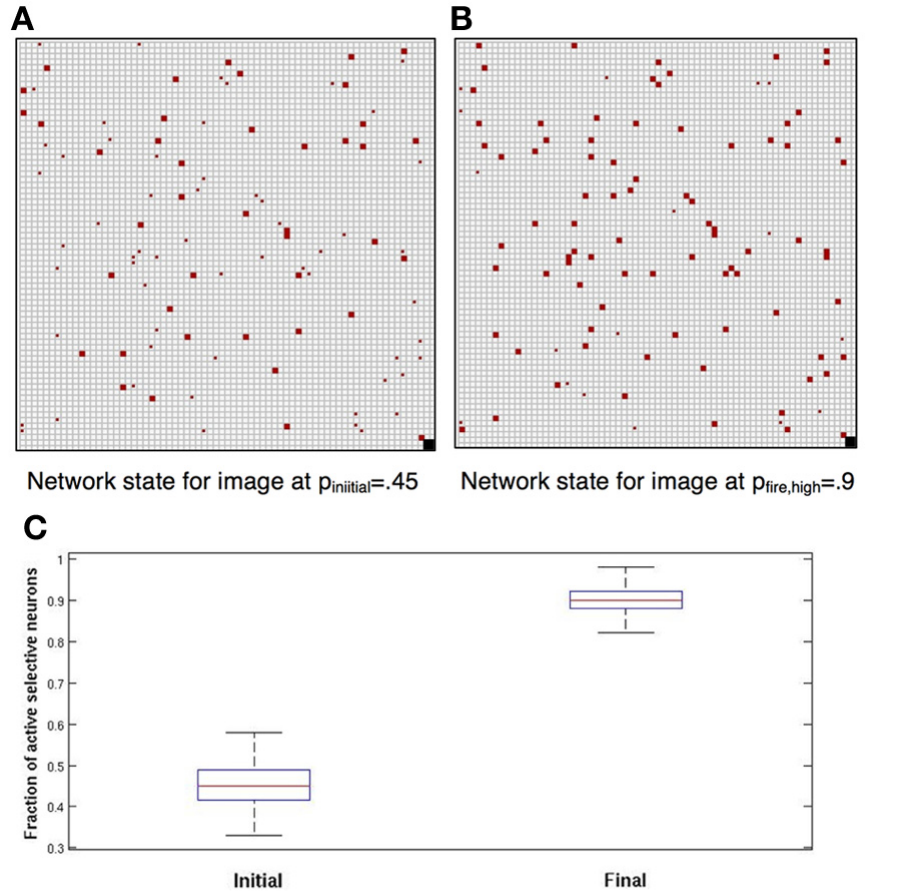
**Completion**. Each square corresponds to a neuron. Blank squares are inactive non-selective neurons. Grid arrangement is just for presentation—network has no topological structure. **(A)** In red the selective neurons of the image. Full squares—active. Small dots—inactive. Initially approximately 45% are active; we set pinitial = 0.45. **(B)** Completion: after network dynamics with *p*_fire_, high = 0.9, approximately 90% of selective neurons are active. **(C)** Box plots showing, for 100 simulations, the distribution of the fraction of active selective neurons, immediately following initial presentation and after completion by network dynamics (red line: median; top and bottom of box: upper and lower quartiles; horizontal lines: quartiles ± 1.5 interquartile range).

Over the past several decades, a large number of delay match to sample (DMS) experiments has been performed with a variety of protocols (Fuster and Alexander, 1971; Miyashita, 1988; Miller and Desimone, 1994; Miller et al., 1996; Yakovlev et al., 2005, 2008, under review); See Figure 1 of accompanying paper (Yakovlev et al., under review). Electro-physiological recordings accompanying these experiments found stimulus selective neurons that fire during the delay period. This activity was seen as reflecting working memory and motivated work on recurrent attractor networks (Amit and Brunel, 1997; Brunel and Wang, 2001; Amit et al., 2003). One distinction between protocols can be found in Miyashita (1988). In one experiment, monkeys were presented with stimulus sequences from a small fixed set of intensively learned images, henceforth denoted *fixed*, and in another they were presented with *novel* images. No persistently firing neurons were detected for *novel* images. Nevertheless, interestingly, the monkeys performed just as well with the novel images. Later, the so-called ABBA modification was proposed (Miller and Desimone, 1994), where among the distractors presented between sample and match, two repeating stimuli were shown, which the monkey learned to ignore. A new protocol, delay match to multiple samples DMMS, was recently introduced (Yakovlev et al., 2005), whereby any image in the sequence could act as the sample, which the monkey needed to remember to signal its repetition at some later point in the trial image sequence. These trials have the form ABCDEC, where C is the sample, which in this trial appears at image 3 and is repeated at image 6. One motivation for this protocol was to control for primacy effects attributed to presentation of the sample as the first image in a sequence. These experiments were performed with fixed sets of images (Yakovlev et al., 2005). In later work the same experiments were performed with novel images (Yakovlev et al., 2008); consistent with the finding of Miyashita (1988) performance was even better than with fixed images. Finally in Yakovlev et al. (under review) we studied monkey behavior when they switched between learned and novel images in the DMMS task, and in particular their ability to handle false positives, signaling an image from one of the previous trials as a repeat. A summary of the different protocols is found in Figure 1 in Yakovlev et al. (under review).

Attractor network models primarily address the problem of maintaining one or more learned images as attractors in a dynamic network of integrate and fire neurons. The model proposed in Brunel and Wang (2001) presented a dichotomy in parameter space between 1—the first pattern staying in working memory and “preventing” other patterns from entering, and 2—the most recent pattern entering working memory and knocking out the previous one. Based largely on this model (Brunel and Wang, 2001), it was found that the network can be tuned to hold multiple images in memory (Amit et al., 2003). A model for a network handling novel images (images seen only once) has been proposed (Yakovlev et al., 2008), and the issues of readout and reset mechanisms are handled there explicitly for the first time, as follows:

Readout refers to the manner in which the network identifies repetition through differential behavior for a repeat image versus a non-repeat. Earlier models for fixed images assumed that if the test image is already in working memory, by definition this means that repetition is detected. No explicit readout mechanism was proposed. With novel images nothing is present in working memory, so repetition readout needs to be achieved otherwise. In Yakovlev et al. (2008) the level of network activity while the stimulus is still present is used. The slight synaptic trace present for a once-seen image induces slightly larger network activity than a never-seen image, although this trace is insufficient to sustain delay activity after the stimulus is withdrawn. Reset refers to a mechanism implemented between trials to erase images from previous trials from working memory. This is necessary to avoid false positives, which occur when the monkey flags an image that appeared in an earlier trial as a repeat.

The models mentioned above employed integrate and fire neurons, and, in the case of fixed images, assumed no overlap between the selective subsets of the stimuli in order to facilitate mean field analysis. Recently, in modeling decision making in the framework of DMS tasks, the different responses to the sample (A) and the repeating distractors (B) were addressed (Engel and Wang, 2011). This difference is achieved by assuming that the initial image has privileged access to a particular network that can retain it in memory, whereas distractor images do not. This model would account for avoidance of repetition detection among distractors, but does not account for the mistakes initially occurring when monkeys learn the ABBA task after performing the original DMS task. The fact that without additional training, monkeys do consider BB a repetition means that the B stimulus *does* reach the network that maintains working memory. Indeed it indicates that false positives would be found in DMS trials if they were systematically tested for. The dichotomy proposed in Brunel and Wang (2001) is also not suitable to describe such a situation. Furthermore, in the experiments described in Yakovlev et al. (2005) the first stimulus has no special status, any stimulus could be repeated, so that any stimulus must have access to the working memory network.

Our goal in the current paper, motivated by previous work (Brunel and Wang, 2001; Amit et al., 2003), is to integrate all these phenomena in a single, parsimonious network model, and account for the different protocols through changes in particular network parameters, assuming that these network parameters are internally modified when confronted with new test circumstances. We concentrate on a network with binary neurons and binary synapses. The learning framework is the same as proposed by Amit and Fusi (1994) with binary synapses undergoing dynamic Hebbian learning, starting from a stationary state that corresponds to the natural assumption that large numbers of patterns have already been learned. However, these authors did not address network dynamics. Indeed dynamics in such binary networks, with threshold and inhibition settings that maximize memory capacity despite significant noise levels, were only addressed recently (Romani et al., 2008; Amit and Huang, 2010). We now show that this single network can account for behavior under these different DMS protocols, including the readout and reset mechanisms. In contrast to the situation with novel images, we propose a readout that is based on a smaller increment in network activity when a pattern present in working memory is shown again. Reset uses the virtual presentation of random unlearned patterns, essentially random background activity, to “wash” out patterns present in working memory. Network parameters (coding level, potentiation and depression rates, threshold, and inhibition) are first set at a baseline level that ensures maximal memory capacity. The different protocols are implemented by appropriately modifying inhibition from baseline, modulating noise level, and in one case modifying depression probability. In particular the form of readout we propose offers a natural explanation for false positives observed in DMMS trials with fixed images, which are not triggered by the previous occurrence of the image in previous trials. To our knowledge this is the first time such an integrated model has been proposed for an array of DMS tasks without artificially limiting the number of learned patterns. Furthermore, the proposed model sheds light on behavior observed when monkeys are switched between different tasks, specifically between DMMS with fixed and novel images.

## Methods and procedures

### Simulated network setup

The network has N binary neurons all with the same threshold θ. The network is fully connected with binary synapses *J*_ij_ = (0/1) leading from neuron *j* to *i*. Images ξ^(*k*)^_*i*_, *i* = 1 … *N*, *k* = 1, … *K*, are sampled randomly with coding level *f*. Specifically for each *k*, with probability *f* the *i*'th neuron is set to be selective for image *k*, i.e., ξ^(*k*)^_*i*_ = 1, independently for each *i*. *S*^(*k*)^ denotes the random set of selective neurons chosen for image *k* whose average size is fN. The threshold is set based on criteria determined in Amit and Huang (2010); Huang and Amit (2011), that guarantee a low expected number of non-selective neurons firing based on the average asymptotic field to such neurons.

#### Learning

Synapses are binary with state *J* = 0 corresponding to the depressed state and *J* = 1 corresponding to the potentiated state. When an image is presented to the network, depressed synapses with active pre and post-synaptic neurons are potentiated with probability *q*_+_ and synapses with active pre-synaptic neuron and inactive post-synaptic neuron are depressed with probability *q*_−_ = αfq_ +_. Learning is initiated from the stationary state of the synaptic matrix, assuming a very large number of patterns has been presented to the network, which randomly assigns about π_+_ = 1/(1 + α) of the synapses to be potentiated.

#### Network dynamics

When image *k* is presented to the network the initial strong visual stimulus is mimicked by activating the units in *S*^(*k*)^ with some level of noise. Each unit is assumed to be on independently with probability *p*_initial_, yielding the initial random active set *A*^(*k*)^_0_ corresponding to this image. This is in addition to whatever other units are already on in the network. The full set of active units at step 0 is denoted *A*_0_. At step *t* there is a current active set of units *A*_*t*_.We randomly choose a neuron for updating from among the *N* neurons in the network. If unit *i* is selected for updating at step *t* its field:

$$h_{\text{i}}=\{\begin{array}{l} \frac{1}{\text{N}}\left(\sum_{\text{j}\in {\text{A}}_{\text{t}}}{\text{J}}_{\text{ij }}-\eta_{\text{inhib}}|{\text{A}}_{\text{t}}|\right)+{\text{C}}^{\text{t}}\text{ if i}\in {\text{A}}_{0}^{\left(\text{k}\right)}\\ \frac{1}{\text{N}}\left(\sum_{\text{j}\in {\text{A}}_{\text{t}}}{\text{J}}_{\text{ij }}-\eta_{\text{inhib}}|{\text{A}}_{\text{t}}|\right)\text{if i}\notin {\text{A}}_{0}^{\left(\text{k}\right)} \end{array}$$

is computed. The second-term represents inhibition and is linear in the number of active neurons. The default value of η_in_hib is the asymptotic probability of a synapse being potentiated, namely π_+_ = 1/(1 + α) (see Amit and Huang, 2010). The third-term *C*^*t*^ is contrast, which mimics the persistence of a strong signal while the stimulus is present, albeit weaker than the initial signal. This is added only to neurons in the initial active image set *A*^(*k*)^ during the presentation period of the stimulus, which corresponds to *T*_contrast_*N* updates. Subsequently contrast is set to zero, corresponding to the period after stimulus presentation, for an additional T_delay_N updates, before an additional stimulus is shown. If the computed field is above threshold, i.e., *h*_i_ > θ we set ξ_i_ = 1 with probability *p*_fire_, otherwise ξ_i_ = 0. The value 1 − p_fire_ corresponds to the noise level in the system. In all models the threshold of the neurons is fixed. The different protocols are obtained by modifying the inhibition level, the noise level *p*_fire_, the contrast level and in one instance the depression rate q_−_.

We note that setting the synaptic states to 0/1 is arbitrary and any two values J_low_, J_high,_ would work with the proper adjustments to the threshold, the contrast and inhibition.

### Readout mechanism for learned images

On initial presentation the number of activated neurons is on average given by ∆_new_ = p_initial_fN, and for an image not active in memory, this would represent the average increase in network activity. On the other hand if the image is currently in working memory, the size of the active set of neurons for that image at iteration t is given by |*A*^(*k*)^_*t*_| ~ p_fire_fN. If the image is presented to the network at step *t* + 1, the increment in network activity would only be from those selective neurons of the image, which are not in *A*^(*k*)^_*t*_ namely on average ∆_old_ = *p*_initial_(1 − *p*_fire_)fN. With any reasonable range of values for p_initial_ and *p*_fire_ we have ∆_old_ ≪ ∆_new_, providing a simple global signal from the network that a match has occurred. The increment threshold for repeat detection is set to be three standard deviations above the mean: $\tau_{\Delta }=\Delta_{\text{old}}+3\sqrt{p_{\text{initial}}\left(1-p_{\text{initial}}\right)\left(1-p_{\text{fire}}\right)fN}$.

### Reset mechanism for learned images

After each trial the network needs to remove active images from memory, otherwise they will produce false positives on subsequent trials. Indeed monkeys need some time to learn to avoid such false positives (Yakovlev et al., under review). The mechanism used here uses the presentation of a sequence of randomly generated images with all parameters held fixed. For any increase in network activity due to the presentation of the random images there is a non-negligible probability that one of the currently active images die out due to inhibition (see also Miller et al., 1996). After several tens of presentations of random images—L_refresh_—with high probability most images residing in working memory will die out. The reset issue is mainly relevant in the repeat detection experiments discussed in section Reset Mechanism for Novel Images. In DMS experiments where the first image holds a privileged position there is little effect of the activity in previous trials.

### Readout mechanism for novel images

A readout mechanism for this setting was proposed (Yakovlev et al., 2008) based on the total level of activity in the network after T_contrast_N updates—before contrast is turned off. The assumption is that the decision is made while the visual stimulus is present or still affecting the network. In this setting we again start from the stationary state of the synapses. Images are presented only once, and the potentiation probability *q*_+_ < 1 is not sufficiently strong to allow these to maintain stable activity in working memory after the contrast period. Nonetheless, even a single presentation of an image leaves a trace in the synaptic matrix and, when presented again, yields a higher activity at the end of the contrast period relative to images that were never before presented to the network. This allows us to determine an absolute activity threshold τ_abs_ that is evaluated after the contrast period of each presentation.

### Reset mechanism for novel images

Since novel images do not reside in working memory attractor activity, the challenge is to eliminate the synaptic trace of the once-seen images at the conclusion of the trial. Eliminating the synaptic trace using the presentation of random images and keeping all parameters the same would require on the order of thousands of image presentations—the memory horizon of familiarity recognition. It seems that the only alternative, as proposed in Yakovlev et al. (2008), is to allow for high depression probabilities during the inter-trial period while presenting random images. In that paper, the authors used a much higher coding level for the random images. Here we did not affect any change to the coding level of the random images, and to maintain simplicity we assume a high depression probability during the entire experiment and set *q*_−_ = 1. In other words, any time a presynaptic neuron is activated and the postsynaptic neuron is off, the synapse is set to 0. Maintaining the depression rate this high does not pose a problem for repetition detection with novel images at small numbers of several tens of images, but would sharply decrease the extent of familiarity recognition relative to the optimal settings.

An alternative to the depression triggered by presenting random images is some form of non-specific long-term depression whereby each synapse independently reverts to state 0 with some probability, *p*_depress_. This may correspond to chemical LTD (see Collingridge et al., 2010), which is not stimulus dependent. In our experiments we chose to implement the former mechanism but in effect they are equivalent. Note that for each seen image, approximately *q*_+_(fN)^2^ synapses are potentiated. When presenting a random image, the probability that each such synapse is between a selective and non-selective neuron is *f*(1 − *f*) so that approximately *Rq*_+_ (*fN*)^2^*f*(1 − *f*) are depressed by the end of R random presentations, which is on average equivalent to *p*_depress_ = *Rf*(1 − *f*).

### Summary of DMS protocols

We summarize the different DMS protocols and the modifications necessary for their implementation in the network:

- Default: Using the default parameters the network adds learned images to working memory. Each image has equal probability of dying out at each step. Readout of repetition with incremental threshold—smaller increment in neural activity.
- DMS: To maintain first image in working memory, contrast time is set to zero (T_contrast_ = 0), and noise level is decreased (*p*_fire,high_ = 0.9) between image presentations, ensuring completion at a high fraction of the first image and preventing its falling out of memory. Upon presentation of a new image the noise level rises again to the default level (*p*_fire_ = *p*_initial_), giving those images present in working memory, which are active at a higher proportion, an advantage over the newly presented images. Thus, with high probability the first image remains in working memory, some additional distractor images may remain there as well. Repetition is read out based on the increment in activity upon stimulus presentation.
- ABBA: Same as DMS but only first image should be maintained in working memory. To ensure this, in addition to decrease in noise level between image presentations, inhibition is slightly increased (η_high_ = 0.34) throughout the experiment.
- Repeat detection with fixed images: Default parameters with 2% increase in inhibition at each step to obtain increase in performance for fixed cue to test distance and longer trials.
- Repeat detection with novel images: high depression rate −q_−_ = 1. This enables elimination of synaptic trace during reset period at the cost of significantly reduced extent of familiarity memory. Readout based on activity after contrast period. Absolute threshold τ_familiar_ = 11.

## Results

Network and synaptic dynamics are described in the Methods and Procedures and summarized in Table 1, with parameters set to maximize memory capacity, and assuming the synaptic states are at stationarity: the network has already been trained on a large number of patterns. In this setting we use simulations to illustrate the modifications that reproduce the different experimental behaviors.

**Table 1:** **Notation formulas and default parameter settings for neural and synaptic dynamics of network and modifications for DMS, ABBA, DMMS trials with novel images**.

*N* = 5000	Number of neurons in network
*J*_*ij*_ = 1/0	State of synapse between neurons *j* and *i*
*f* = 0.02, *fN* ~100	Coding level—average number of selective neurons in each pattern
*q*_+_ = 1.0 (0.3)	Synaptic potentiation probability for fixed images, (novel images)
*q*_−_ = 3*fq*_+_ = 0.059, (*q*_−_ = 1.0)	Synaptic depression probability for fixed images, (novel images)
Potentiation: If ξ _*j*_ = 1, ξ_*i*_ = 1, *J*_*ij*_ = 0 ⇒ *J*_*ij*_ ↗ 1, with probability *q*_+_	
Depression: If ξ_*j*_ = 1, ξ_*i*_ = 0, *J*_*ij*_ = 1 ⇒ *J*_*ij*_ ↘ 0, with probability *q*_−_	
*p*_initial_ = 0.45	Probability a selective neuron is on at stimulus presentation
*p*_fire_ = 0.45	Probability neuron fires if field is above threshold
θ = 0.004	Neuron threshold
π_+_ = 0.254	Stationary probability for synapse to be potentiated
η_inhib_ = π_+_ = 0.254, (0.34)	Inhibition factor, (for ABBA trials)
*C* = θ	Contrast - level of external input when stimulus is present
$h_{i}=\frac{1}{N}\left(\sum_{j\in A_{t}}J_{ij}-\eta_{\text{inhib}}|A_{t}|\right)+C_{i}^{t}$	Field of neuron *i* at iteration *t*. If *h*_*i*_ > θ neuron is on with probability *p*_fire_. *C*^*i*^_*t*_- contrast at neuron *i* at step *t*. Only at level *C* if neuron is an initially activated selective neuron and *t* < *NT*_contrast_
$\tau_{\Delta }=p_{\text{initial}}(1-p_{\text{fire}})fN+3\sqrt{p_{\text{initial}}(1-p_{\text{initial}})(1-p_{\text{fire}})fN}=36$	Activity increment threshold, below which repeat is called for fixed images
*T*_contrast_ = 0, (3)	Multiple of N updates with contrast for fixed images, (novel images)
*L*_refresh_ = 40	Number of random images presented during reset period
τ_abs_ = 11	Absolute activity threshold above which repeat is called for novel images
*p*_fire, high_ = 0.9	Low noise level for DMS trials after each new stimulus

### Default setting

Images are learned with the default parameters (Table 1), starting from a stationary synaptic matrix of about one in four potentiated synapses. These images are then shown in random order and network dynamics is computed. Upon image presentation, units selective to the presented image are moved into their active state, with a noise-dependent probability *p*_initial_, but irrespective of their state before image presentation. This probability represents the uncertainty involved in the actual appearance of the stimulus, and the processing it undergoes in the brain until reaching the recurrent network. It is not an internal network parameter and is therefore fixed and not modulated. A number of network iterations is then performed with “contrast,” i.e., assumed further external input to these image-selective units corresponding to a period of continued image presence, albeit at a lower level of input than that due to the initial signal. These two stages of input may be seen as corresponding to the initial transient and later steady state responses ubiquitous in sensory physiology. Then external input is removed and dynamics continues with no “contrast,” driven only by the internal fields generated by the network. At all times there is a level of inhibition on all units, which is proportional to the total number of active units. If the net input to a unit (by synapses from other active units and “contrast” from presentation of the image to which it is selective, less general inhibition) is above a fixed threshold, the unit is moved to its active state with a probability *p*_fire_. Note that *p*_initial_ and *p*_fire_ are inversely related to the corresponding noise levels. To reiterate, *p*_initial_ refers to the uncertainty in the signal arriving at the network and remains fixed, whereas *p*_fire_ refers to the uncertainty in above threshold neural firing in the recurrent network and can be modulated. Several important phenomena are observed.

#### Large memory capacity

The network can stably recall a large number of learned images. It has been shown that a network with 5000 neurons has a capacity of approximately 200 learned images and a network of 100,000 neurons has a capacity of approximately 60,000 (Amit and Huang, 2010).

#### Completion

The network reliably performs image completion. If the noise level during the dynamics is lower than the initial stimulus noise at presentation, i.e., *p*_fire_ > p_initial_ the network quickly evolves from an initial proportion of about *p*_initial,_ active selective neurons to a higher proportion of about *p*_fire_ active selective neurons. This is demonstrated in Figure 1. This fundamental property of recurrent network models provides for elimination of noise and convergence toward a more abstract representation of the stimulus. In Figure 1, each grid square corresponds to a neuron (the grid-like arrangement is used for ease of presentation; the network is fully connected, without topological structure). Blank squares are inactive neurons, not selective to the current image. Full red squares are active selective neurons and smaller red dots show inactive selective neurons.

#### Sustained activity following stimulus removal

If no additional learned stimuli are presented to the network, following image-presentation completion, the attractor state corresponding to the stimulus will remain active for long periods and be robust to noise level changes (see below). However, this is not the natural situation since learned stimuli are ubiquitous and will constantly be appearing. This leads to the following.

#### Sustained activity for multiple images

**Figure 2:**
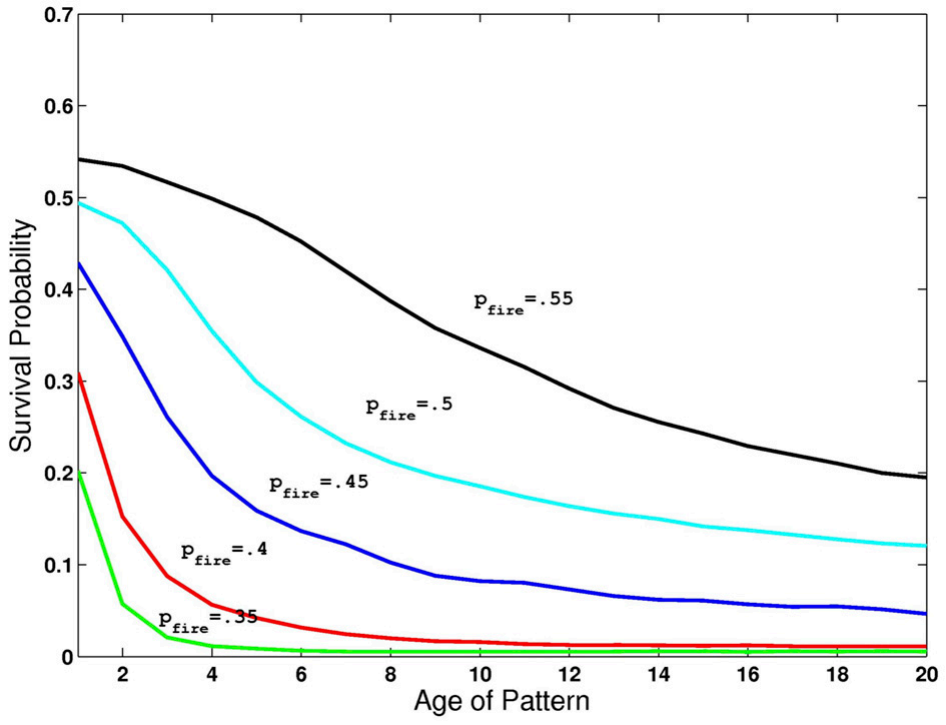
**Retention of multiple images in recurrent activity**. Probability of image maintaining recurrent activity as a function of its age for different levels of *p*_fire_ and all other parameters at default levels given in Table 1.

The network can simultaneously maintain several images in working memory, that is, synapses among neurons selective to the same image are sufficiently strong to maintain activity even in the presence of many other active neurons, and the accompanying higher level of inhibition. However, since inhibition grows with the number of active neurons, as additional learned images are presented to the network, older ones gradually die out. This depletion of image representations occurs mainly during the period that each new image is presented. At this time, the contrast being applied to the newest image reduces its probability of dying out, giving it an advantage over other images currently resident in memory. On the other hand, when this newest image is no longer being presented, all image representations have equal probabilities of dying out due to inhibition. Thus, the probability that an image has survived decreases with age. In Figure 2 we show the probability of an image staying in memory as a function of its age (in units of additional images presented) counting back from the latest one presented. All network parameters are at the default setting and we allow *p*_fire_ to vary between 0.35 and 0.55. Note the different rates of decay of survival probability as a function of age.

### Readout—incremental threshold

**Figure 3:**
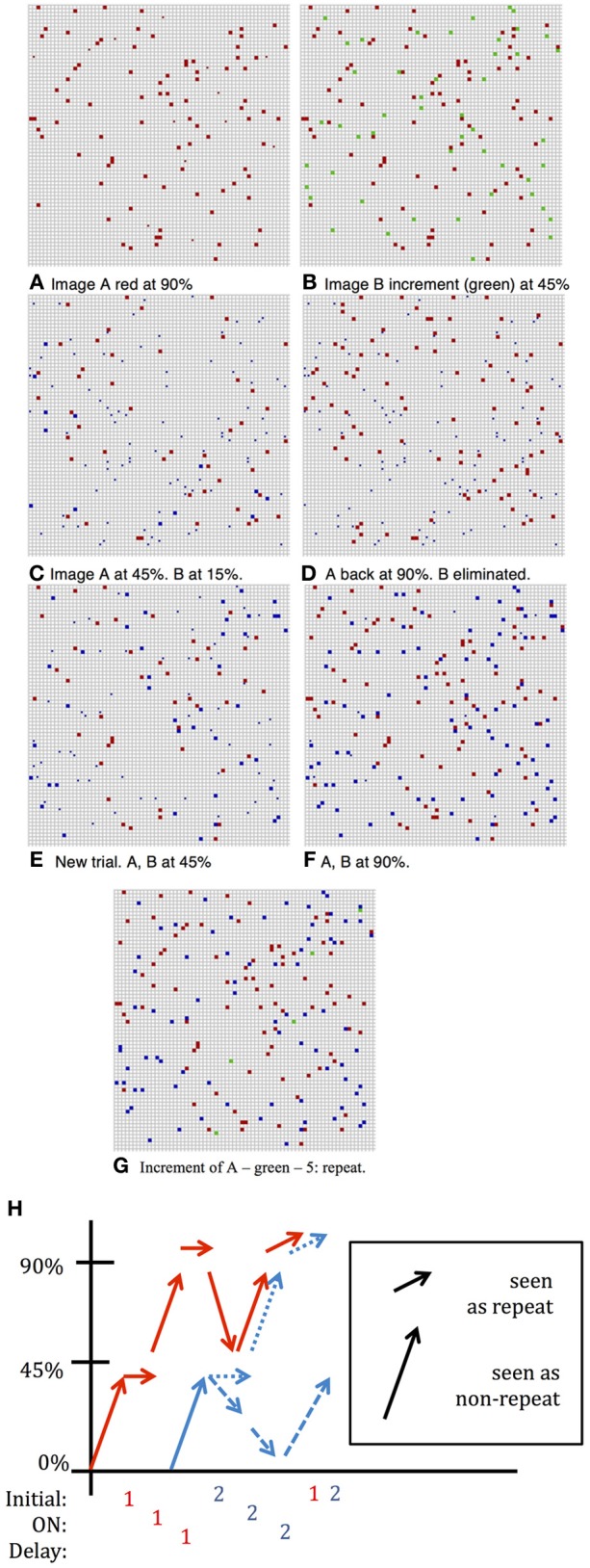
**DMS Simulation**. Full squares—active selective units, small dots inactive selective units; color indicates neuron's preferred image; fN ~ 100. **(A)** Situation following presentation of first image and several iterations for completion by network dynamics with *p*_fire,high_ = 0.9. **(B)** Second image added with *p*_initial_ = 0.45, the noise level of the incoming stimulus. First image (red) still sustained at low noise level: *p*_fire,high_ = 0.9. Increment in green units is large, ~ 45—response is: no repetition seen. **(C)** After a few iterations at higher noise level: *p*_fire_ = 0.45, first image (red) sustained at 45% and second image (blue) sustained at about 15% and will die out **(D)** following inter-trial iterations, only first image completes to 90%. **(E)** New Trial. After presentation of second image (and despite first image having completed to 90%) and after a few iterations at *p*_fire_ = 0.45, first image (red) and second image (blue) sustained at about 45%—both survive **(F)** Completion of both images to 90% level. Had second image been presented the increment would be small and a repeat flagged. **(G)** First image presented again to network. Increment (shown in green)—5 units: repeat detected. **(H)** Summary of activity for different stimulations described above.

The question of how a network codes for a “match,” i.e., a repetition of a previously presented image, has not received much attention in the literature. With fixed images the increase in network activity on presentation of the match is *smaller* relative to presentation of a non-match image. This is illustrated in Figure 3. Panel **(B)** shows the increment, in green, when an image is presented which is not currently sustained in the network and thus has little overlap with the image currently in memory. Recall that at the moment of the new stimulus presentation, images in memory are sustained at the low noise level. In contrast panel **(G)** shows the increment, in green, when the first image is repeated with both the first and second images in memory. We suggest that the fact that the increment is smaller is used by the network as an indication that the last image presented is a match to the originally presented cue image. This decreased increment signal does not require that the network has any knowledge of which images are in working memory nor that the network directly compare the neurons that are selective of the test image to the active neurons in the network.

### DMS trials

To strengthen and maintain activity of the neurons representing the first presented image that acts as the cue for which a match is to be found, we enhance the probability, *p*_fire_, of firing for neurons with above-threshold activation, i.e., we reduce the internal noise level, except during presentation of subsequent images. This periodic modulation of noise level (high when an image is presented, low otherwise) allows units representing the first image to remain active with high probability, and may also prevent other images from remaining active. The two possibilities are illustrated in Figure 3. As in Figure 1, the first image appears with about 45% of its selective units activated, and after several network iterations at the lower noise level, (i.e., higher probability of above threshold firing), the image representation completes to about 90% active (Figures 1B, 3A). When the second image is presented the initial stimulus noise is high and the representation is at approximately 45%, whereas the first image representation is still at ~90% (Figure 3B). Then the network works again at the higher noise level, which reduces the first image representation from 90% to about 50%, leaving the newer image representation at about 15% (Figure 3C). The initial disadvantage of the new image sometimes suffices so that despite reverting to lower noise, with the first image representation growing back to 90%, the second newer image representation dies out completely (Figure 3D). In these cases, a repetition of the second image will induce a large response increment and thus not be perceived as a repetition. Sometimes, however, both images arrive at representations of about 45% active selective units (as seen in Figure 3E, first image represented by red units, second image by blue). In this case, when the network reverts to the low noise level during the delay period, both images reach 90% activity (Figure 3F). In this case, when the first (or equally when the second) image is repeated and the increment (in green) is small, this signals a repeat (Figure 3G). Thus, in this second case, where the second image representation does not die out, it, too, would trigger the repeat signal if it were presented again, failing the ABBA protocol test. The extent of such false positives has not usually been tested in standard DMS experiments. These changes in incremental activity upon stimulation are summarized in Figure 3H.

**Figure 4:**
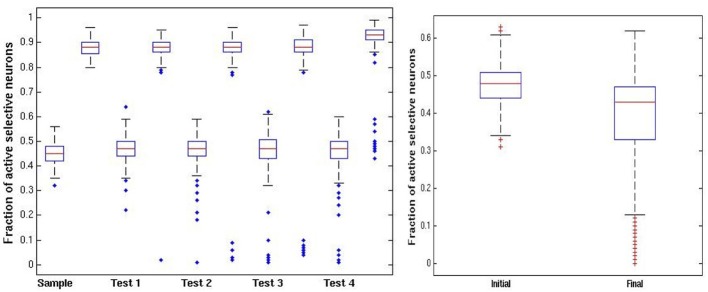
**DMS trials. Left panel**: distribution of fraction of active selective units for the sample (first) image at each stage of the trial (100 simulations). Each stage shows a pair of boxplots. The first shows the resulting activity after the network is run in the high noise regime *p*_fire_ = 0.45. The second shows the resulting activity after the low noise regime *p*_fire,high_ = 0.9. **Right panel:** fraction of selective units active on intermediate test images. First boxplot shows the initial activity upon presentation of the image. The second boxplot shows the activity after running the network in the high noise regime, starting with the high activity level of the sample image.

In Figure 4 we illustrate some statistics of these dynamics. For the initial sample we show a boxplot of the distribution of activity of selective neurons after each epoch of high noise and low noise regimes over 100 simulations (central red line: median; upper and lower lines of the box: first and third quartiles; two horizontal lines quartiles ±1.5 interquartile range; additional points—outliers.). The epochs start upon presentation of an image with the network running at high noise level. The sample image activity oscillates stably between the 45% range to the 90% range (left panel). Test images are presented while the sample image is at high activity after an epoch of low noise level, putting them at a disadvantage during the high noise epoch immediately following their presentation. In the right panel of Figure 4 we show the fraction of active selective neurons for the intermediate test images immediately upon presentation and after the high noise epoch. There is a reduction in activity of the selective neurons in the second boxplot, some die out, whereas others maintain activity at a very wide range around the 45% median, see also the two examples of Figure 3.

### ABBA trials

**Figure 5:**
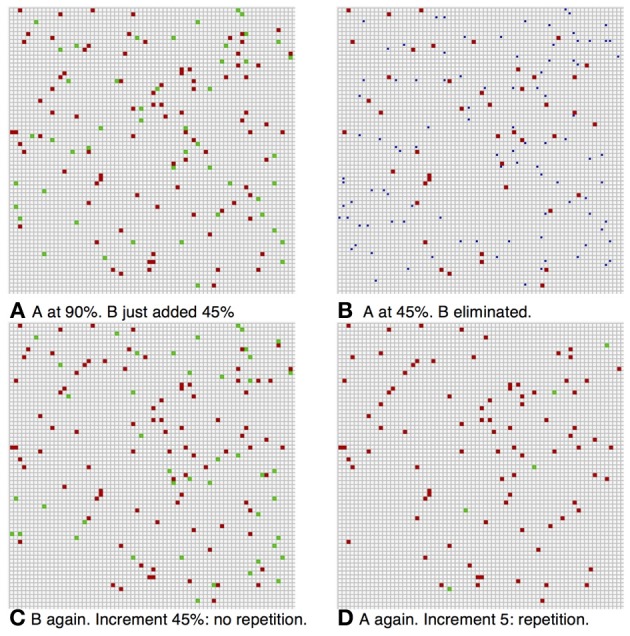
**ABBA simulation**. Same parameters as Figure 3 with inhibition increase **(A)** Image B added at *p*_initial_ = 0.45, first image (red) still at 90%. Increment in green ~45%—no repeat. **(B)** After iterations at *p*_fire_ = 0.45, η_inhib,high_ = 0.34, image A (red) at ~45% activity, image B died out. **(C)** Image A completed to 90% level, image B repeated (BB) increment in green ~45%—no repeat. **(D)** Second presentation of image B dies out, image A completes to 90%, and after repeat of image A—small increment of 5 units (green)—repeat.

The above protocol will succeed if the only repeated image shown in the sequence is the initial sample. If a test stimulus is repeated and happens to have stayed in memory it would trigger a low increment match response. It was reported that when monkeys were initially trained on the standard DMS task they *did* signal repeats of the test images (BB) (Miller and Desimone, 1994) as predicted by our simulation. Additional training was needed for them to learn to avoid the B repeats. In our setting this type of error is avoided by increasing inhibition to level η_inhib,high_ above the baseline level η _inhib_ that was originally set to optimize the capacity of the network. The increased inhibition raises the competitive advantage of the first image, which is at 90% activity level relative to the incoming images that start out at 45% activity level. An example of the dynamics in an ABBA trial is shown in Figure 5. Due to the higher inhibition, the second image, which in 5(A) activated about 45 selective neurons (in green), has nearly died out in 5(B). The increment on repeated presentation of the second image in 5(C) is around 45 since very few of its selective neurons remain active. In contrast when image A is repeated in 5(D), since its representation is at a high activity level of around 90 selective neurons, the increment is particularly small—about 10 neurons.

**Figure 6:**
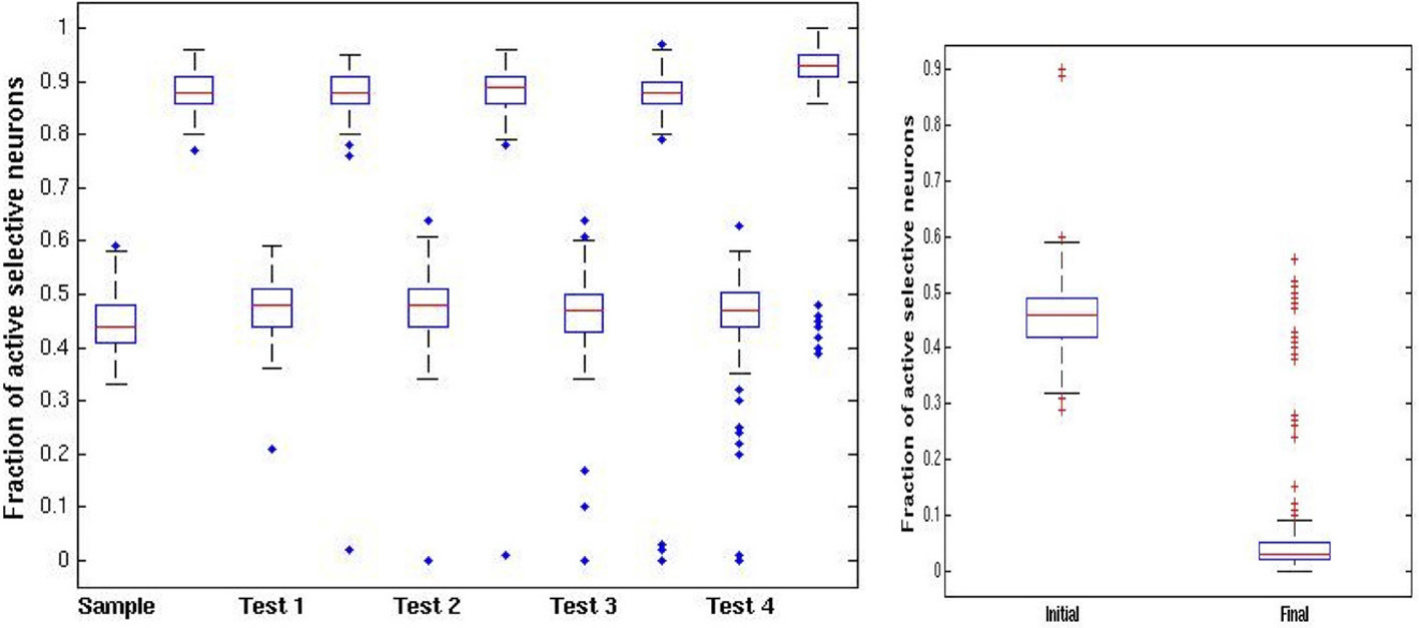
**ABBA trials. Left panel:** distribution of fraction of active selective units for the sample (first) image at each stage of the trial (100 simulations). Each stage shows a pair of boxplots. The first shows the resulting activity after the network is run in the high noise regime *p*_fire_ = 0.45. The second shows the resulting activity after the low noise regime *p*_fire,high_ = 0.9, **Right panel:** fraction of selective units active on intermediate test images. First boxplot show the initial activity upon presentation. The second boxplot shows the activity after running the network in the high noise regime starting with the high activity level of the sample image. In contrast to the DMS case, the increased inhibition causes most test images to die out.

In Figure 6 we show the same statistics as in Figure 4 for the new ABBA protocol. Note the much greater decrease in activity of non-match test patterns after presentation right panel of Figure 6.

In both standard DMS and ABBA trials we set the contrast period T_contrast_ = 0, since contrast breaks the symmetry in favor of the newest stimulus; the opposite of what we need to maintain the activity of the first image. This is equivalent to a faster decrease of the strength of the sensory stimulus or lower steady state condition. The opposite is required for detection of repetition with novel images, where contrast is an essential ingredient (see section Summary of DMS Protocols).

### DMMS trials, fixed images

The natural model for DMMS is to have all presented images stay in working memory (Amit et al., 2003). Modulation of noise and inhibition is not needed. The performance plots in Yakovlev et al. (2005) and Figure 2 of Yakovlev et al. (under review) show that the larger the distance between the sample and its repetition, the worse the performance. This is consistent with the default network setting where older images gradually die out and new ones have an advantage due to the contrast period. The deterioration of performance as the lag increases seems to indicate that the method employed by the brain is indeed that of maintaining the reverberating activity of attractors corresponding to the different images. One twist is that for the same lag between sample and repeat, performance improves the longer the trial. This is achieved in the simulations using a gradual decrease in inhibition at each image presentation, and may correspond to a growing expectation for a repeat.

**Figure 7:**
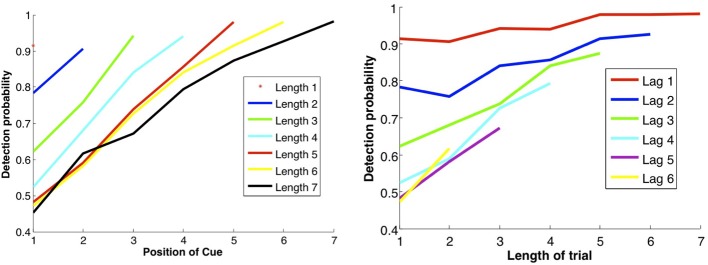
**DMMS performance. Left panel**: each curve corresponds to a trial of different length, i.e., number of samples before the match, as shown in the legend. Success rates as a function of the location of the sample in the trial: the later the sample position and the shorter the trial length, the shorter the lag between sample and repeat, and the less time for the attractor to dissipate, so the better the performance. **Right panel:** each curve corresponds to a fixed lag between sample and repeat, as a function of trial length. Unexpectedly, the longer the trial, the better the performance, perhaps due to growing expectation—and vigilance—for a match, modeled by gradually decreasing inhibition. Note that the data points in the two panels are the same; they are just connected differently to produce different curves.

**Figure 8:**
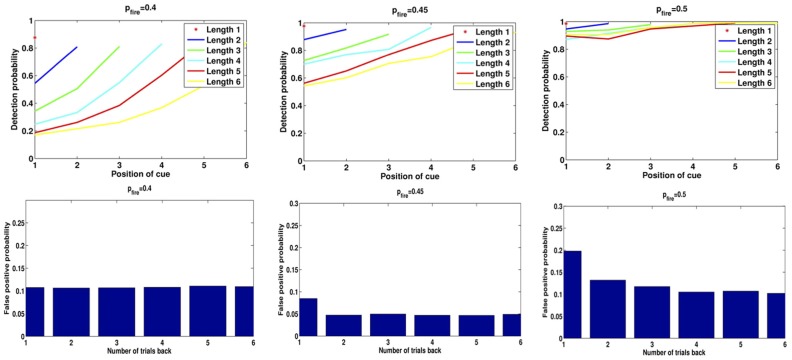
**Detection rates and false positives for different levels of *p*_fire_**. **Left:** *p*_fire_ = 0.4, **Middle:** *p*_fire_ = 0.45, **Right:** *p*_fire_ = 0.5. **Top:** detection rates, **Bottom:** false positive probability for repetition of a pattern from 1 to 6 trials back.

In Figure 7 we show the results of running the network with the default parameters and a 2% decrease in inhibition after each stimulus. The network was trained on 20 images and trials were of length 1–6 with repetitions of images at all locations, with multiple random orderings. When the repeated image is presented the incremental activity is measured and compared to threshold. Results are similar to those reported in Yakovlev et al. (2005, under review), both for the success rates as a function of stimulus position for different trial lengths and the success rates for fixed lags at different trial lengths. To achieve results corresponding to experiments, both in terms of detection probabilities and in terms of false positive rates, we varied one parameter: *p*_fire_. The top row of Figure 8 shows the detection probabilities for *p*_fire_ = 0.4, 0.45, 0.5, respectively. With *p*_fire_ = 0.4 the survival probability of the patterns is very low (see also Figure 2) and detection probabilities are far lower than those in the experiments. With *p*_fire_ = 0.5 the survival probabilities of the patterns is very high and detection probabilities are far higher than those in the experiments. Interestingly the value of *p*_fire_ that yields the best match to experimental results in terms of detection probabilities also yields the best match to the experimental results in terms of the false positive rates, see the bottom row of Figure 8.

#### Reset mechanism and false positives

After each trial the network needs to remove active images from memory, otherwise they produce false positives on subsequent trials. Monkeys need to learn to achieve this since, as in the default network setting, it is not the standard behavior. In simulations reset is achieved by pseudo-presenting a sequence of randomly generated patterns (see Methods and Procedures).

One form of False Positive (FP) is then a result of the readout mechanism and is due to random fluctuations in the size of the increment. Even if an image is not in memory there is a non-negligible probability that the size of the set of selective neurons initially activated for that image is less than increment threshold for repeat detection, τ_Δ_. Thus, there is no need to use “pop-in” events for learned images as proposed in Yakovlev et al. (2005), which in fact were never observed in our simulations. The second and more prevalent form of FP is due to the failure of the reset mechanism to erase from memory all images from previous trials. With the default parameter settings we obtain the following numbers consistent with experimental numbers (Yakovlev et al., 2005, under review): 5.6% of all test presentations led to FPs. Of these, 4.9% are due to the second mechanism, i.e. the image remained in memory from previous trials. The remaining 0.7% are due to random fluctuations. In Figure 8 we show for three different values of *p*_fire_ (0.4, 0.45, 0.5) the detection rates as in Figure 7, and the false positive rates due to repetition of patterns from 1 to 6 trials back. Compare the plots for *p*_fire_ = 0.45 to data in Figure 2 in Yakovlev et al. (under review).

### Repeat detection, novel images

A lingering puzzle in the context of DMS experiments is successful performance with entirely novel stimuli (Yakovlev et al., 2008, under review). It is unlikely that novel stimuli are learned to the point that they can sustain delayed activity in working memory. Indeed it was already noticed that neurons do not exhibit delay activity with novel stimuli (Fuster and Alexander, 1971). This implies that a different readout mechanism is detecting the repetition.

#### Readout mechanism with novel images

The images are presented once and the synaptic modifications are unable to sustain delay activity. Nonetheless, some trace is present in the synaptic matrix and, when presented again, the stimulus yields higher activity at the end of the contrast period, relative to images that were never seen before. Detecting this higher activity allows us to stably identify repeats over vast numbers of images presented only once. That is, having seen thousands of images, and not seen thousands more, we (and monkeys) can identify a currently presented image as belonging to the seen-once or never-seen group. It has been demonstrated (Romani et al., 2008) that with values of *q*_+_ on the order of 0.1–0.3, and depression rate *q*_−_ = α*fq*_+_ set to its optimal level, once-seen images are not sustained in delay activity, however, repeat detection is possible over many thousands of images. Success probability, based on the proposed readout mechanism, is thus constant over many tens if not hundreds of images, consistent with behavioral observations (Yakovlev et al., 2008), and in contrast to performance with a fixed set of images. Attractor working memory is thus a *liability* for simple repeat detection. However, anything more complex, such as the ABBA task would be impossible with novel images, as there is no way to suppress the effect of the repeat of the B sample.

#### Comparison of readout mechanisms

The readout mechanism proposed for novel images differs from that of a fixed set of images that trigger reverberating activity of an attractor. The signal telling the system which readout to use could be presence or absence of network activity prior to stimulus presentation. The former means presence of images in working memory and readout is based on activity *increment* (small increment means repeat); the latter means no images are in working memory and readout is based on *absolute* activity level, (greater activity indicates repeat).

#### Reset mechanism

**Figure 9:**
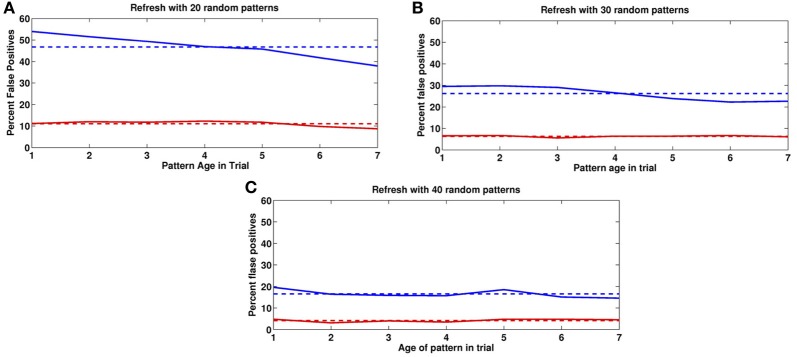
**False positives for novel images**. False positive rates as a function of age in past trials. Blue: previous trial. Red: two trials back. Horizontal lines correspond to means. **(A)** 20, **(B)** 30, **(C)** 40 random image presentations in the Inter-trial period.

In Figure 9 we show the impact of different numbers of random image pseudo-presentations (20, 30, 40) in the inter-trial period and a very high depression rate of *q*_−_ = 1. In all three experiments the mean activity of a previously presented image after the end of the contrast period, was around 16 (with SD = 4) selective neurons, compared to the average of 45 at noise level *p*_fire_ = 0.45. The mean activity of an image that was never seen before was 8 with the same SD. We set the absolute readout threshold to provide just over 90% detection rate so that τ_abs_ = 11. This threshold yields the false positives shown in Figure 6 for the three experiments. As the number of inter-trial pseudo-presentations of random images increases, the synaptic trace of the once seen images decreases, as does the FP rate. Panel **9(C)**, with 40 inter-trial presentations yields FP rates similar to those described behaviorally (Romani et al., 2008; Yakovlev et al., 2008).

### Switching protocols

Other experiments provide further challenges for modeling DMS (Yakovlev et al., under review). Monkeys are transitioned from one protocol to another and behavior is recorded. In Experiment I, monkeys B and T start out with the fixed images protocol, achieve performance similar to that presented in section DMMS Trials, Fixed Images, and are then transitioned in Experiment II to novel images. This is compared to monkeys D and L who start out with novel images and are transitioned to fixed images. Monkeys BT learn to control FPs in Experiment I, since they are penalized for declaring repeats that aren't from the same trial. Monkeys DL do not learn about FPs because all images are novel. In a following test stage, an occasional “catch” image is inserted, which is a repeat of a novel image from a previous trial. With high probability this image triggers a response from the monkey. Indeed FP rates for monkeys in group DL are at 90% for images from the preceding trial.

When monkeys BT are transitioned to novel images the FP rate drops rather quickly to levels comparable with Experiment I. This is in contrast to monkeys DL who start out with novel images (Yakovlev et al., under review). Furthermore, monkeys BT exhibit an increase in detection rates originally observed with monkeys DL for novel images. A possible explanation is that monkeys BT have already developed the strategy for avoiding FPs (section Reset mechanism and false positives) and only need to increase depression rates to eliminate synaptic traces, as opposed to the easier task of eliminating sustained activity. Furthermore, as no sustained activity is present, the monkey uses absolute readout. With these modifications network performance matches that shown in the experiments.

On the other hand, monkeys DL encounter the challenge of FPs for the first time in Experiment II. Images are gradually learned together with the reset mechanism, and the fixed images protocol is applied.

## Discussion

Recognition memory is widely believed to be composed of at least two functionally distinct processes: recollection and familiarity. Recollection is defined as the ability to accurately describe an event or object that was encountered before. Familiarity, on the other hand, only involves signaling that an event was encountered in the past, but without the ability to recall any details. The relation between recollection and familiarity is unclear. According to the dual-process view, recollection and familiarity are based on different neurological substrates (Mandler, 1980; Jacoby et al., 1993; Yonelinas, 1994; Brown and Aggleton, 2001) and different networks (Bogacz et al., 1999, 2001). In contrast, single-process models assume that recollection and familiarity reflect strong and weak memories, respectively, and that these two processes differ only quantitatively (Rotello et al., 2005; Squire et al., 2007; Wixted, 2007; Cohen et al., 2008). In providing a unified model for DMS tasks, in a single network, for both novel and fixed images, we are promoting the single-process model, where the difference between these two types of memory stems directly from the number of presentations of the images during learning. An image presented only once leaves a weak synaptic trace and cannot sustain recurrent activity, as opposed to an image presented several times. Both types of image can be simultaneously stored in the network. The difference in the strength of the synaptic trace has a major impact on the dynamics of the network upon presentation of the image in the testing stage. As a result, the readout mechanism must be different. For familiarity detection with novel images that do not reside in attractor working memory, we use the absolute network activity, for recollection with fixed images we use the increment in activity, since the image is assumed to be currently in working memory. An important advantage of this modeling approach is parsimony—one mechanism can account for multiple phenomena.

The ability to learn, forget, and maintain recurrent activity in binary networks with simple Hebbian learning offers a rich computational setting to explore a range of experimental phenomena. Although the temporal evolution of neural responses is absent, the ensemble behavior of these discrete networks is similar to those with more elaborate neural modeling (Brunel and Wang, 2001; Amit et al., 2003; Romani et al., 2008; Yakovlev et al., 2008). On the other hand, since the simulations are fast and can be performed for very large networks on a simple PC, this is an exciting test bed for many interesting scenarios.

Our model offers a number of predictions that can be experimentally tested. We mention a few:

- Noise modulation. This was a necessary component for keeping the first stimulus in memory despite the initiation of multiple distractors throughout the trial.
- Modulation of inhibition. We found it necessary to increase inhibition in ABBA trials to remove distractors that may repeat. Can inhibition levels be tested and compared for a monkey trained initially on a DMS task and then transitioned to an ABBA task?
- Increase in depression rates. This was required to reset the synaptic trace in the repeat detection for novel images. Can this be measured together with its effect on memory retention?
- Random image presentation for reset. Is there evidence of higher firing activity between trials?
- Upon a third transition of monkeys DL from novel back to fixed, the network may maintain a high depression rate and have difficulties learning the new fixed set. Detection performance would then be at the higher level observed with novel images.

The transition from one protocol to another is an interesting framework for testing our hypotheses regarding particular changes in network parameters through careful analysis of behavior and electrophysiology during the transition.

### Conflict of interest statement

The authors declare that the research was conducted in the absence of any commercial or financial relationships that could be construed as a potential conflict of interest.
